# A non-invasive preoperative prediction model for predicting axillary lymph node metastasis in breast cancer based on a machine learning approach: combining ultrasonographic parameters and breast gamma specific imaging features

**DOI:** 10.1186/s13014-024-02453-2

**Published:** 2024-05-27

**Authors:** Ranze Cai, Li Deng, Hua Zhang, Hongwei Zhang, Qian Wu

**Affiliations:** 1 Department of Neurosurgery, Zhongshan Hospital (Xiamen), Fudan University, Xiamen, Fujian Province 361006 China; 2 Department of General Surgery, Shanghai Public Health Clinical Center, Shanghai, 201508 China; 3 Department of General Surgery, Zhongshan Hospital, Fudan University, Shanghai, 200032 China; 4 Department of General Surgery, Fudan University Affiliated Huadong Hospital, Shanghai, 200040 China

**Keywords:** Breast neoplasms, Axillary lymph node, Machine learning, Ultrasonography, Breast specific gamma image

## Abstract

**Background:**

The most common route of breast cancer metastasis is through the mammary lymphatic network. An accurate assessment of the axillary lymph node (ALN) burden before surgery can avoid unnecessary axillary surgery, consequently preventing surgical complications. In this study, we aimed to develop a non-invasive prediction model incorporating breast specific gamma image (BSGI) features and ultrasonographic parameters to assess axillary lymph node status.

**Materials and methods:**

Cohorts of breast cancer patients who underwent surgery between 2012 and 2021 were created (The training set included 1104 ultrasound images and 940 BSGI images from 235 patients, the test set included 568 ultrasound images and 296 BSGI images from 99 patients) for the development of the prediction model. six machine learning (ML) methods and recursive feature elimination were trained in the training set to create a strong prediction model. Based on the best-performing model, we created an online calculator that can make a linear predictor in patients easily accessible to clinicians. The receiver operating characteristic (ROC) and calibration curve are used to verify the model performance respectively and evaluate the clinical effectiveness of the model.

**Results:**

Six ultrasonographic parameters (transverse diameter of tumour, longitudinal diameter of tumour, lymphatic echogenicity, transverse diameter of lymph nodes, longitudinal diameter of lymph nodes, lymphatic color Doppler flow imaging grade) and one BSGI features (axillary mass status) were selected based on the best-performing model. In the test set, the support vector machines’ model showed the best predictive ability (AUC = 0.794, sensitivity = 0.641, specificity = 0.8, PPV = 0.676, NPV = 0.774 and accuracy = 0.737). An online calculator was established for clinicians to predict patients’ risk of ALN metastasis (https://wuqian.shinyapps.io/shinybsgi/). The result in ROC showed the model could benefit from incorporating BSGI feature.

**Conclusion:**

This study developed a non-invasive prediction model that incorporates variables using ML method and serves to clinically predict ALN metastasis and help in selection of the appropriate treatment option.

**Supplementary Information:**

The online version contains supplementary material available at 10.1186/s13014-024-02453-2.

## Background

Breast cancer has become the most prevalent cancer worldwide with an estimated 2.3 million new cases in 2020 [1], the most common route of breast cancer metastasis is through the axillary lymphatic network. Therefore the status of the axillary lymph nodes (ALN) plays an important role in tumour staging, postoperative therapy and tumour prognosis. An accurate assessment of the ALN burden before surgery can avoid unnecessary axillary surgery, consequently preventing surgical complications such as lymphedema, sensory abnormalities, and limitation of upper limb movement [2, 3]. The possibility of exempting axillary surgery in early breast cancer have been widely explored in several clinical trials [4–6]. However, with the limited randomized, multicenter clinical trials and strict inclusion criteria, proper selection of axillary surgeries for patients who fail to meet the criteria has become a priority of many clinicians. Previous studies [7, 8] have attempted to develop models to assess the ALN burden individually to facilitate clinical decision making.

Ultrasonography (US), mammography and magnetic resonance imaging (MRI) are three commonly used imaging modalities for detecting breast cancer, with all techniques relying on the anatomic differences between cancer and normal breast parenchyma [9]. Mammography is the golden standard in cancer screening. However, it remains limited as it only provides information about the anterior axilla when assess the ALN status and its sensitivity is significantly reduced in women with dense breast tissue [10]. MRI shows its superiority in terms of sensitivity and specificity for breast cancer, but some patients are unable to undergo MRI evaluation due to implantable devices, body habitus, renal insufficiency, and claustrophobia. US has the advantage of being convenient, radiation-free, inexpensive and non-invasive. However, in view of the variations between different operators, US has a high false positive rate which limits the clinical application. Therefore, supplementary methods of breast screening must be found. Breast-specific gamma imaging (BSGI) is a recently emerging molecular breast imaging modality that can detect breast cancers at the sub-centimetre level and at various breast tissue densities, with a sensitivity estimate of 90–96% [11, 12]. In BSGI, a radiotracer such as Technetium-99 m Sestamibi is injected into the patient’s bloodstream and the breast is visualized using a special camera. The radiotracer uptake is commensurate with blood flow and mitochondrial activity within tumour cells [13], which enables us to diagnose breast cancer by distinguishing the biological behaviour between tumour cells and normal cells. In addition, The BSGI presents an excellent specificity for the diagnosis of ALN metastasis [14]. This prompted us to wonder the possibility of incorporating BSGI, a modality with accuracy comparable to MRI, to improve the accuracy of ALN status prediction. We reviewed the previous literature and found none exploring this possibility yet.

Machine learning (ML) is an emerging tool for cancer prediction and prognosis that is making significant contributions in different cancer fields [15, 16]. It is a learning process capable of providing excellent accuracy through a continuous mechanical learning approach using techniques like decision trees (DTs), artificial neural networks (ANN), and support vector machines (SVM), ultimately developing prediction tools which in some cases outperform traditional statistical modeling.

Thus, the aim of our study was to employ ML-based statistical methods to select variables from non-invasive preoperative modalities, such as BSGI and US, ultimately establish a prediction model to assess the ALN burden, which can guide clinicians for better choice of cancer treatment options for different patients.

## Methods

### Patients

**Fig. 1:**
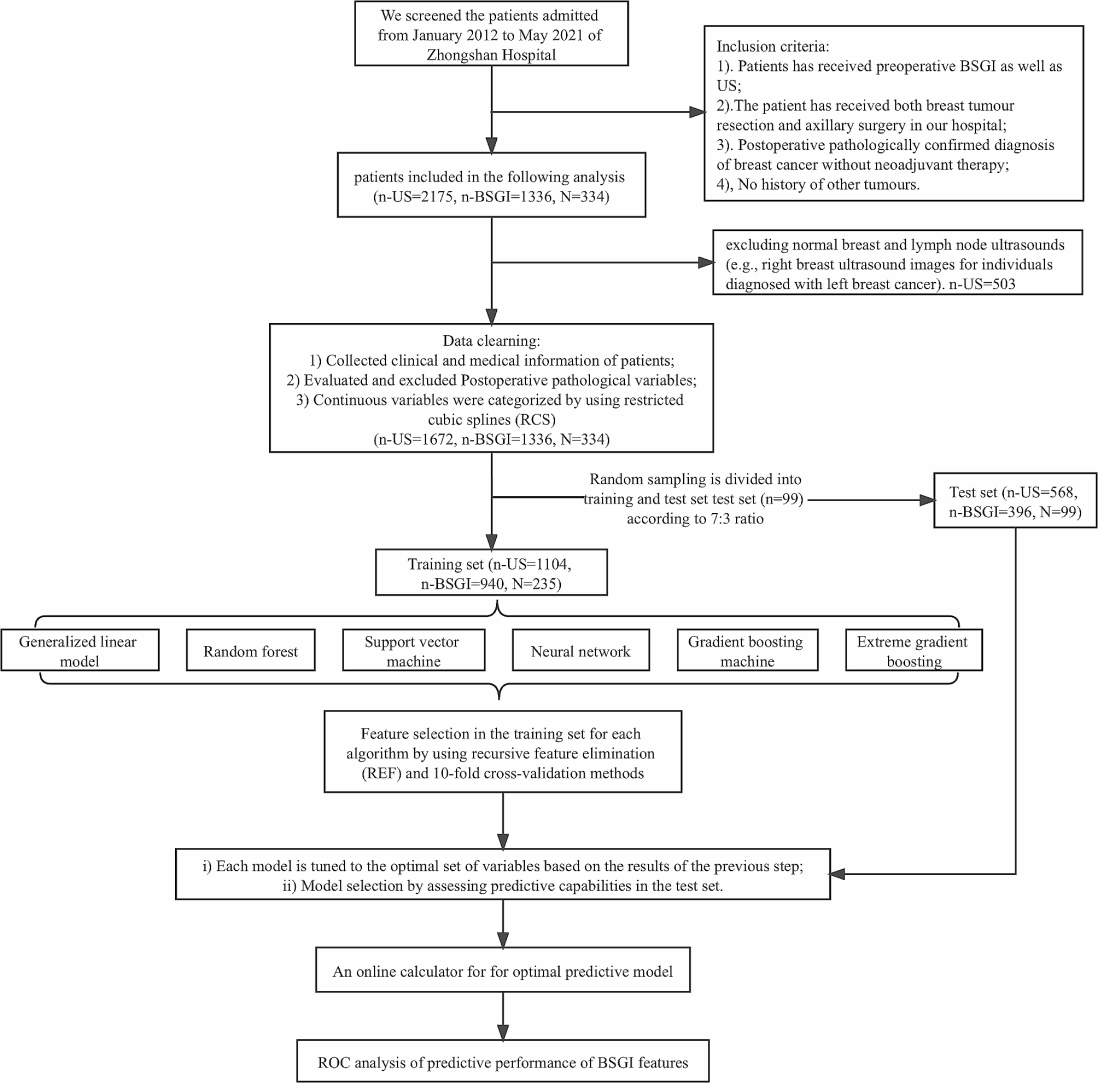
Working flow of this study

The clinical data of patients who underwent surgery between January 2012 and May 2021 at Zhongshan Hospital (an affiliate of Fudan University) were collected consecutively and analyzed retrospectively. The inclusion criteria were: 1. Patients has received preoperative BSGI as well as US; 2. The patient has received both breast tumour resection and axillary surgery in our hospital; 3. Postoperative pathologically confirmed diagnosis of breast cancer without neoadjuvant therapy; 4. No history of other tumours. Normal breast and lymph node ultrasounds imagines were excluded. The ethical approval of this study was granted by ethics committee of Zhongshan Hospital. All methods were carried out in accordance with relevant guidelines and regulations. Zhongshan Hospital Ethics Committee waived the need of informed consent from patients since it was a retrospective study. The working flow of our study was showed in Fig. 1.

### BSGI and ultrasonographic results

Patients underwent BSGI (Dilon 6800; Dilon Technologies) at high-resolution and a small field-of-view. Imaging was performed 10–15 min after the intravenous administration of 740 MBq Technetium-99 m Sestamibi (GMS Pharmaceutical Co. Ltd) through an antecubital vein contralateral to the suspicious breast side. Craniocaudal (CC) and mediolateral oblique (MLO) images of both breasts were obtained. A low-energy general-purpose collimator was used, with a photopeak focused at 140 keV with a symmetric 10% window. The acquisition time was approximately 6 min per image and a value of 100 000 counts per image was defined as the minimum range.

**Fig. 2:**
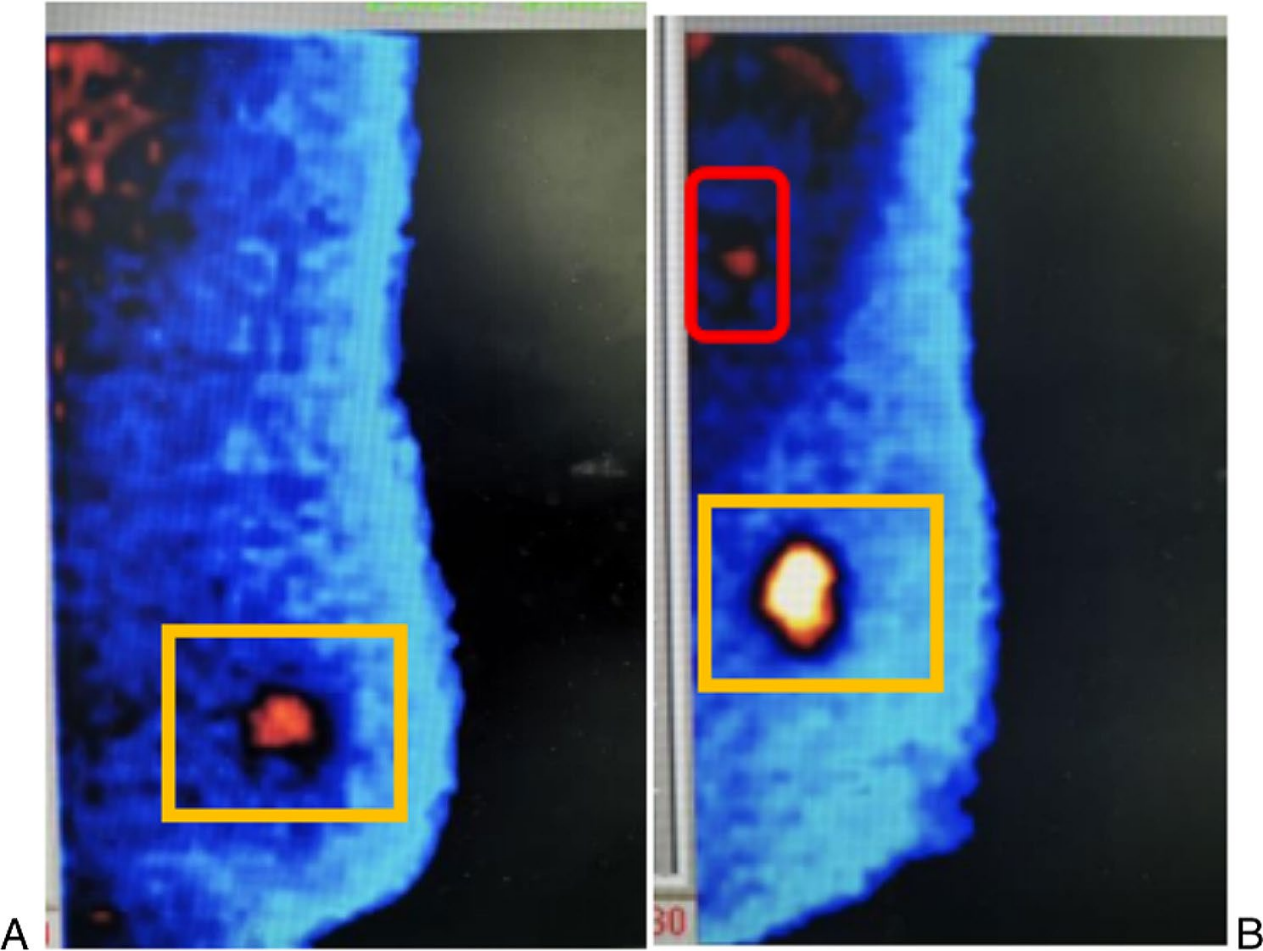
An example of breast-specific gamma imaging analysis. (**A**) showed a left-sided breast cancer without axillary lymph node metastasis, the yellow rectangle showed the uptake of Technetium-99 m Sestamibi in breast. (**B**) showed a left-sided breast cancer with axillary lymph node metastasis, the red circle showed a positive axillary mass

Two experienced nuclear medicine physicians analyzed the images and were blinded to the patients’ clinical information and pathological results. According to the 2010 guidelines of the Breast Imaging Reporting and Data System (BIRADS) of the Society and Nuclear Medicine and Molecular Imaging [17], lesions with homogeneous and small patchy uptake were considered to be negative, lesions with patchy uptake, mild focal uptake and definite focal uptake were considered to be positive. The tumour-to-normal lesion ratio (TNR) was calculated by dividing the maximal pixel counts of the tumor lesion by that of normal background breast tissue on both CC and MLO view. A positive axillary mass was considered to be patchy, mild focal and definite focal uptake of Technetium-99 m Sestamibi in axilla. An example of BSGI imagines analysis was showed in Fig. 2.

**Fig. 3:**
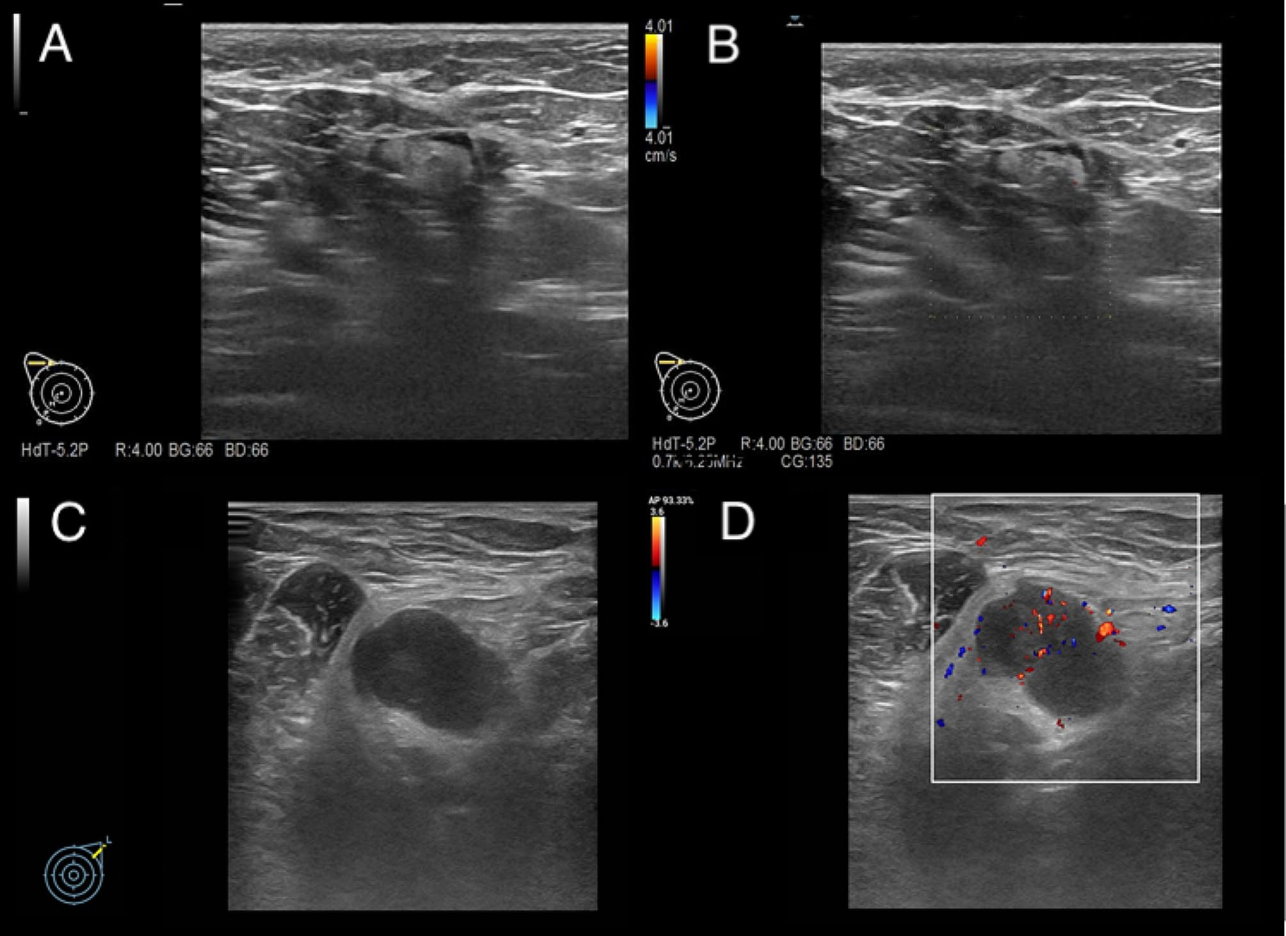
An example of ultrasonographic lymph node imagines analysis. (**A**) showed an ultrasound image of the right axilla exhibiting no signs of lymph node metastasis. (**B**) showed a color Doppler flow imaging (CDFI) image of the right axilla, indicating the absence of lymph node metastasis. (**C**) showed an ultrasound image of the left axilla showcasing lymph node metastasis, featuring a 24.0*16.0 mm mass identified as a lymph node. (**D**) showed a CDFI ultrasound image of lymph node metastasis in the left axilla, revealing discernible colored blood flow within the affected lymph node

The ultrasonographic images were obtained using an HDI 5000 scanner (Philips Medical Systems) and analyzed by 2 experienced operators. According to Adler’s method [18], the degree of blood flow signal within the breast carcinomas and axillary lymph nodes was subjectively classified into 1 of 4 levels: absent (grade 0), minimal (grade 1), moderate (grade 2), or abundant (grade 3). The echogenicity was classified as cystic (no echo), hypoechoic, isoechoic, hyperechoic and mixed echoic. When a mass showed echogenicity minimally less than that of subcutaneous fat, it was defined as hypoechoic. An example of ultrasonographic imagines analysis was showed in Fig. 3.

### Data collection

The collected clinical and medical information of patients included the patients’ gender, age, breast tumour location, BSGI features (tumour TNR and axillary mass status), postoperative pathological features (estrogen receptor (ER) status, proliferation index (Ki-67), progesterone receptor (PR) status, Her-2 overexpression, lymphovascular invasion (LVI), Scarff-Bloom-Richardson (SBR) grade, T stage, N stage, infiltration depth, histologic type, molecular subtype, and multifocality) and ultrasonographic parameters of tumour and axillary lymph nodes (sizes, echogenicity, margin, lymph node hilum status, color Doppler flow imaging (CDFI) grade, and resistance index (RI)).

### Statistical analysis

Clinical and pathological variables associated with the risk of lymph node metastasis were assessed on the basis of their clinical importance and predictors identified in previously published articles [19, 20]. Categorical variables, such as patients’ gender, tumor location, axillary mass status (BSGI), tumor echogenicity, tumor margin, tumor CDFI, lymphatic echogenicity, absence of lymph node hilum, lymphatic CDFI, infiltration depth, histologic type, SBR grade, ER status, PR status, Ki-67, Her-2 overexpression, molecular subtype, LVI, multifocality, T stage, and N stage, were reported as integers and proportions. On the other hand, continuous variables, including patients’ age, tumor TNR (CC), tumor TNR (MLO), transverse diameter of tumor, longitudinal diameter of tumor, tumor RI, transverse diameter of lymph nodes, and longitudinal diameter of lymph nodes, were reported as means with standard deviations. The association between clinicopathological characteristics and ALN status was analyzed using X2 test or t-test as appropriate. Collinearity for all explanatory variables were assessed using correlation matrix and plausible interaction terms were also tested. To relax the assumption of a linear relationship between continuous predictors and the risk of ALNs metastasis, continuous variables (such as the patients’ age, tumor TNR (CC), tumor TNR (MLO), transverse diameter of tumor, longitudinal diameter of tumor, tumor RI, transverse diameter of lymph nodes, and longitudinal diameter of lymph nodespatient) were converted into categorical variables after valuation using restricted cubic splines (RCS) [21]. Patients were randomly sampling into the training and test sets by ratio 7 : 3. Six machine learning (ML) methods were trained in, including generalized linear model (GLM), random forest (RF), support vector machines (SVM), neural network (NNET), gradient boosting machine (GBM), extreme boosting machine (XGB) [22–24]. To select the strongest predictive variables, recursive feature elimination (REF) was used for each algorithm. To avoid overfitting in training, the best hyper-parameter for ML models was 10-fold cross-validation. Comparison of ML methods performance, the best classification model was select. Based on the best-performing model, we created an online calculator that can make predictor in patients easily accessible to clinicians. Finally, the receiver operating characteristic (ROC) analysis was used to assess the role of BSGI feature in this prediction model [25]. All statistical analyses were determined using the R software (version 3.6.3, http://www.r-project.org). The R packages “caret”, “rms”, “glmnet”, “randomForest”, ‘nnet’, “e1071”, “kernlab”, “pROC”, “gbm”, and “xgboost” were used. The “shiny” package was used for web application. We used t-test for continuous variables and chi-square test for classified variables. A two sided *P* < 0.05 was considered statistically significant.

## Results

### Demographic and clinicopathological characteristics

A total of 1672 ultrasound images and 1336 BSGI images from 334 patients were screened between January 2012 to May 2021. Patients were grouped into two groups according to the presence/absence of axillary lymph metastasis. The clinicopathological characteristics between the metastasis and non-metastasis patients were shown in Table 1. The median patient age was 58 years, and 52.1% patients’ tumour located on upper-outer quadrant. Patients showed no difference in gender, age, location, tumour echogenicity, tumour margin, tumour CDFI grade, lymph node hilum status, histological type, multifocality, ER, PR, or Her-2 status. However, a higher value of tumour TNR either on CC or MLO view, a positive axillary mass on BSGI, a lower lymphatic echogenicity, a longer transverse or longitudinal diameter of tumour or lymph node, a higher CDFI grade of lymph node, a higher SBR grade, a higher proliferation index (Ki-67), a deeper infiltration, Her-2 positive subtype and the presence of tumour LVI each showed a higher likelihood for ALN metastasis (*P* < 0.05).

**Table 1:** Differences of clinicopathological characteristics between the patients with and without axillary lymph metastasis

	Metastasis (*n* = 131)	Non-Metastasis (*n* = 203)	All (*n* = 334)	*P*value
**Gender**				
male	1 (0.8%)	2 (1.0%)	3 (0.9%)	
female	130 (99.2%)	201 (99.0%)	331 (99.1%)	1
**Age**	56.8 (11.9)	58.5 (11.5)	57.8 (11.7)	0.182
**Tumour location**				
UOQ	68 (51.9%)	106 (52.2%)	174 (52.1%)	
LOQ	27 (20.6%)	34 (16.7%)	61 (18.3%)	
UIQ	23 (17.6%)	42 (20.7%)	65 (19.5%)	
LIQ	12 (9.2%)	14 (6.9%)	26 (7.8%)	
central	1 (0.7%)	5 (3.5%)	6(2.3%)	0.410
**BSGI features**				
**Number of BSGI images**	524 (39.2%)	812 (60.8%)	1336 (100%)	
**Tumour TNR (CC)**	3 (1.3)	2.7 (1.3)	2.8 (1.3)	0.007
**Tumour TNR (MLO)**	2.9 (1.4)	2.5 (1.2)	2.6 (1.3)	0.002
**Axillary mass**				
negative	73 (55.7%)	180 (88.7%)	253 (75.7%)	
positive	58 (44.3%)	23 (11.3%)	81 (24.3%)	< 0.001
**Ultrasonographic features**				
**Number of ultrasound images**	654 (39.1%)	1018 (60.9%)	1672 (100%)	
**Tumour echogenicity**				
hypoechoic	129 (98.5%)	196 (96.6%)	325 (97.3%)	
isoechoic	1 (0.8%)	0 (0.0%)	1 (0.3%)	
mixed	1 (0.8%)	7 (3.4%)	8 (2.4%)	0.137
**Transverse diameter of tumour (mm)**	24.1 (10.3)	20.7 (9.8)	22 (10.1)	0.001
**longitudinal diameter of tumour (mm)**	15.1 (6.4)	13.7 (6.4)	14.2 (6.4)	
**Tumour margin**				
regular	1 (0.8%)	3 (1.5%)	4 (1.2%)	
irregular	130 (99.2%)	200 (98.5%)	330 (98.8%)	0.943
**Tumour CDFI**				
no signal (0)	16 (12.2%)	31 (15.3%)	47 (14.1%)	
spot (I)	7 (5.3%)	20 (9.9%)	27 (8.1%)	
linear (II)	64 (48.9%)	89 (43.8%)	153 (45.8%)	
abundant (III)	44 (33.6%)	63 (31.0%)	107 (32.0%)	0.372
**Tumour RI**	0.7 (0.3)	0.6 (0.3)	0.7 (0.3)	
**Lymphatic echogenicity**				
cystic	26 (19.8%)	90 (44.3%)	116 (34.7%)	
hyperechoic	26 (19.8%)	83 (40.9%)	109 (32.6%)	
hypoechoic	76 (58.0%)	29 (14.3%)	105 (31.4%)	
isoechoic	2 (1.5%)	1 (0.5%)	3 (0.9%)	
mixed	1 (0.9%)	0 (0.0%)	1 (0.4%)	< 0.001
**Transverse diameter of lymph nodes (mm)**	13.6 (10)	6.4 (6.2)	9.2 (8.7)	< 0.001
**longitudinal diameter of lymph nodes (mm)**	8 (6.6)	3.1 (3)	5 (5.3)	< 0.001
**Absence of lymph node hilum**				
no or not described	126 (96.2%)	201 (99.0%)	327 (97.9%)	
yes	5 (3.8%)	2 (1.0%)	7 (2.1%)	0.17
**Lymphatic CDFI**				
no signal (0)	62 (47.3%)	187 (92.1%)	249 (74.6%)	
spot (I)	10 (7.6%)	7 (3.4%)	17 (5.1%)	
linear (II)	38 (29.0%)	6 (3.0%)	44 (13.2%)	
abundant (III)	21 (16.0%)	3 (1.5%)	24 (7.2%)	< 0.001
**Lymphatic RI**	0.1 (0.3)	0 (0)	0 (0.2)	< 0.001
**Pathological features**				
**Infiltration depth**				
in situ	0 (0.0%)	13 (6.4%)	311 (93.1%)	
infiltrative	128 (97.7%)	183 (90.1%)	10 (3.0%)	
other	3 (2.3%)	7 (3.4%)	13 (3.9%)	0.01
**Histologic type**				
ductal	124 (94.7%)	186 (91.6%)	310 (92.8%)	
lobular	4 (3.1%)	6 (3.0%)	10 (3.0%)	
other	3 (2.3%)	11 (5.4%)	14 (4.2%)	0.379
**SBR grade**				
not described	5 (3.8%)	42 (20.7%)	47 (14.1%)	
I	3 (2.3%)	10 (4.9%)	13 (3.9%)	
II	53 (40.5%)	81 (39.9%)	134 (40.1%)	
III	70 (53.4%)	70 (34.5%)	140 (41.9%)	< 0.001
**Estrogen receptor status**				
negative	30 (22.9%)	59 (29.1%)	89 (26.6%)	
positive	101 (77.1%)	144 (70.9%)	245 (73.4%)	0.264
**Progesterone receptor status**				
negative	44 (33.6%)	80 (39.4%)	124 (37.1%)	
positive	87 (66.4%)	123 (60.6%)	210 (62.9%)	0.338
**Proliferation index (Ki-67)**				
< 14%	17 (13.0%)	51 (25.1%)	68 (20.4%)	
≥ 14%	114 (87.0%)	152 (74.9%)	266 (79.6%)	0.011
**Her-2 overexpression**				
negative	89 (67.9%)	156 (76.8%)	245 (73.4%)	
positive	42 (32.1%)	47 (23.2%)	89 (26.6%)	0.095
**Subtype**				
Luminal A	13 (9.9%)	39 (19.2%)	52 (15.6%)	
Luminal B	88 (67.2%)	105 (51.7%)	193 (57.8%)	
Her2 positive	19 (14.5%)	26 (12.8%)	45 (13.5%)	
Triple negative	11 (8.4%)	33 (16.3%)	44 (13.2%)	0.009
**lymphovascular invasion**				
no	95 (72.5%)	190 (93.6%)	285 (85.3%)	
yes	36 (27.5%)	13 (6.4%)	49 (14.7%)	< 0.001
**Multifocality**				
no	127 (96.9%)	199 (98.0%)	326 (97.6%)	
yes	4 (3.1%)	4 (2.0%)	8 (2.4%)	0.791
**T stage**				
Tis	0 (0.0%)	13 (6.4%)	13 (3.9%)	
T1	57 (43.5%)	116 (57.1%)	173 (51.8%)	
T2	68 (51.9%)	68 (33.5%)	136 (40.7%)	
T3	6 (4.6%)	6 (3.0%)	12 (3.6%)	< 0.001
**N stage**				
N0	0 (0.0%)	203 (100%)	203 (60.8%)	
N1	70 (53.4%)	0 (0%)	70 (21.0%)	
N2	38 (29.0%)	0 (0%)	38 (11.4%)	
N3	23 (17.6%)	0 (0%)	23 (6.8%)	< 0.001

UOQ: Upper-outer quadrant; LOQ: Lower-outer quadrant; UIQ: Upper-inner quadrant; LIQ: Lower-inner quadrant; BSGI: breast specific gamma image; TNR: tumour-to-normal lesion ratio; CC: craniocaudal; MLO: mediolateral oblique; CDFI: color Doppler flow imaging; RI: resistance index; SBR grade: Scarff-Bloom-Richardson grade

### Prediction model and factors selection

**Fig. 4:**
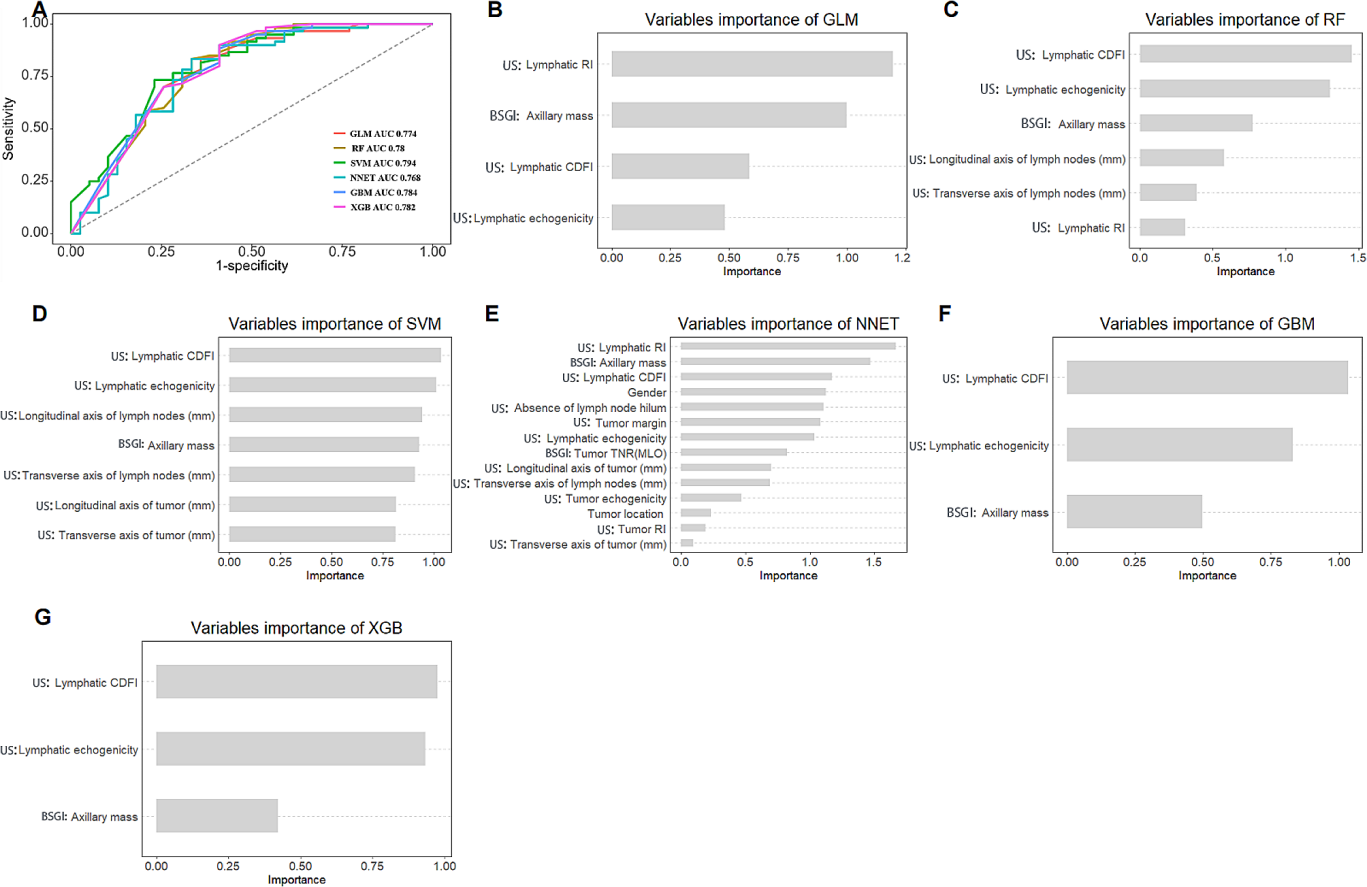
Model selection. (**A**) The line graph shows the predictive values of each model in test set. (**B-G**) The relative weights of selected variables in each model. GLM: generalized linear model; RF: random forest; SVM: support vector machine; NNET: neural network; GBM: gradient boosting machine; XGB: extreme boosting machine; RI: resistance index; CDFI: the color Doppler flow imaging grade; TNR: tumour-to-normal lesion ratio; MLO: mediolateral oblique; BSGI: breast specific gamma image; US: Ultrasonography

Patients were randomly divided into the training and test groups (group ratio 7 : 3). The training set comprised 1672 ultrasound images and 940 BSGI images sourced from 235 patients. Conversely, the test set consisted of 568 ultrasound images and 396 BSGI images collected from 99 patients. All explanatory variables were turned into categorical form and the cutoffs of continuous variables after RCS processing. All pathological characteristics were excluded since we intended to build a non-invasive model. No statistical difference of variables between training set and test set was found in Table S1. We use six ML methods and combine them with REF to select the optimal combination of variables within each algorithm (Figure S1). The optimal sets of variables for each algorithm in the training set were selected, these variables sets were then passed into each ML method to tune and validated the model in the test set. In Fig. 4A; Table 2, the model of SVM showed best predictive ability in the test set (AUC = 0.794, sensitivity = 0.641, specificity = 0.8, PPV = 0.676, NPV = 0.774 and accuracy = 0.737).

**Table 2:** Predictive performance comparison of different machine learning models in the test set

ML model	Accuracy	Kappa	Sensitivity	Specificity	PPV	NPV	AUC
GLM	0.758	0.453	0.487	0.933	0.826	0.737	0.774
RF	0.778	0.508	0.564	0.917	0.815	0.764	0.78
**SVM**	0.737	0.445	0.641	0.8	0.676	0.774	**0.794**
NNET	0.768	0.507	0.667	0.833	0.722	0.794	0.768
GBM	0.737	0.435	0.59	0.833	0.697	0.758	0.784
XGB	0.737	0.435	0.59	0.833	0.697	0.758	0.782

ML: machine learning; GLM: generalized linear model; RF: random forest; SVM: support vector machine; NNET: neural network; GBM: gradient boosting machine; XGB: extreme boosting machine; PPV: Positive predictive value; NPV: Negative predictive value; AUC: the area under the ROC curve.

### Relative importance of variables in machine learning algorithms

The relative weights of each optimal variables set in each model were shown in Fig. 4B-G. The best parameters of each model for their optimal variables were shown in Table S2.

Although obvious differences were shown in the importance of variables among those ML algorithms, factors including lymphatic CDFI grade, lymphatic echogenicity and BSGI axillary mass status rank top three without fail. However, if only these three variables were selected it would affect the classification results of the ML algorithms. The importance of high-ranking variables in the SVM model is arranged as follows in a descending order: lymphatic CDFI grade, lymphatic echogenicity, longitudinal diameter of lymph nodes, axillary mass status, transverse diameter of lymph nodes, longitudinal diameter of tumour, transverse diameter of tumour.

### Web-based calculator

**Fig. 5:**
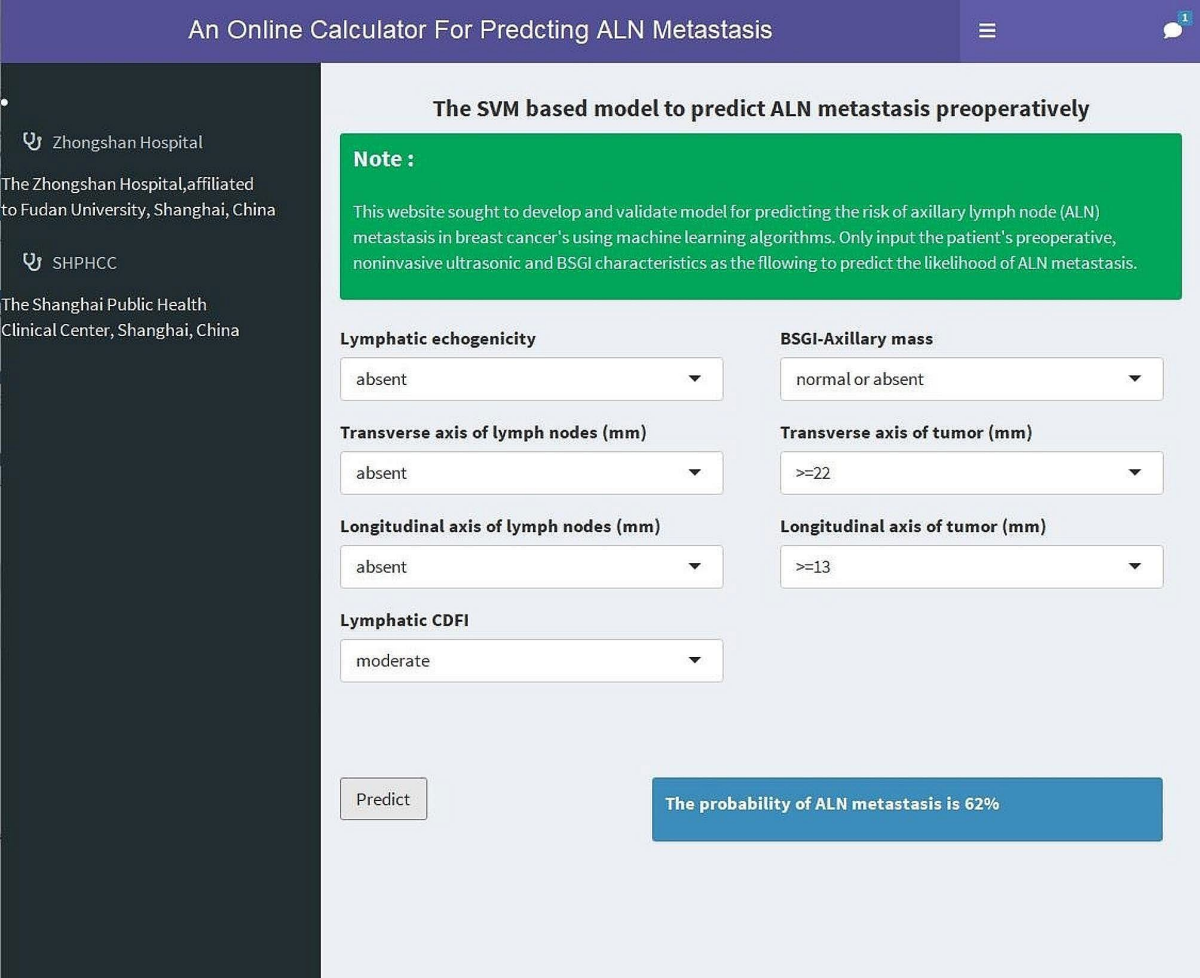
An online calculator for predcting ALN metastasis. SVM: support vector machine; ALN: axillary lymph node; CDFI: the CDFI grade of lymph node; BSGI: breast-specific gamma imaging

An online calculator based on the best-performing model was established for clinicians to predict patients’ risk of ALN metastasis by simply inputing preoperative clinicopathological variables (https://wuqian.shinyapps.io/shinybsgi/) (Fig. 5).

### Clinical application evaluation of BSGI

**Fig. 6:**
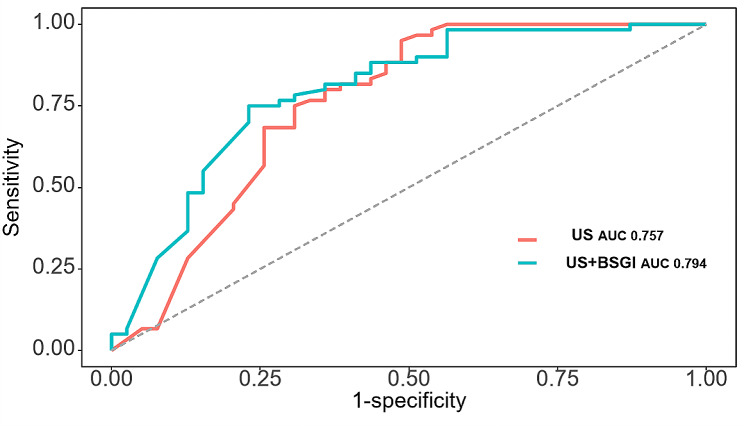
Evaluation of the model effectiveness with and without the BSGI features by ROC curves. US: Ultrasonography; BSGI: breast-specific gamma imaging; AUC: area under the curve

In Fig. 6, a ROC analysis showed that using the final SVM model with BSGI features provided additional benefit from only ultrasonographic features. Thus, We believed the inclusion of BSGI was important to improve preoperative prediction of ALN metastasis in breast cancer patients, especially when lymph node metastases cannot be identified by ultrasonography. This can facilitate early clinical intervention and thus support personalized postoperative cancer rehabilitation.

## Discussion

The shift toward less invasive local treatment for axillary sugery is inevitable with the increased use of systemic and radiation therapy. The NSABP b-32 [4] trial found that sentinel lymph nodes biopsy (SLNB) alone without further ALN dissection (ALND) is an appropriate therapy for the targeted patients and has become a routine surgical procedure. Although the incidence of complications after SLNB is relatively low compared to ALND [26], given that dissection of the mammary lymphatic network is unavoidable during SLNB, patients undergoing SLNB may similarity experience subsequent complications such as limited range of motion, lymphoedema, pain, and sensory defects [2, 3]. It prompted us to ponder whether we could exempt axillary surgery for patients with a particularly small probability of metastasis, whether we could develop a completely non-invasive model to predict the probability of ALN metastasis to improve the patients’ quality of life.

In this study, we attempted for the first time to combine BSGI features and ultrasonographic parameters to predict ALN metastasis, six different ML methods were analysed and compared to derive optimal solutions for the combination of variables, and the SVM model was found to have the best performance. Six ultrasonographic parameters (transverse diameter of tumour, longitudinal diameter of tumour, lymphatic echogenicity, transverse diameter of lymph nodes, longitudinal diameter of lymph nodes, lymphatic CDFI grade) and one BSGI features (axillary mass status) were eventually incorporated into the final model. The final AUC value obtained for the test set was 0.794, which showed a comparatively high prediction ability. While SVM exhibited the highest AUC values, we observed closely clustered AUC values among the six ML methods, ranging from 0.76 to 0.79. In a study by Zhu et al. [27], six ML methods were applied to predict postoperative central lymph node metastasis in T1-T2 thyroid cancers, yielding AUCs between 0.69 and 0.75. In another study by Wu et al. [28], seven ML methods were used to predict central lymph node metastasis in thyroid cancer, with AUCs ranging from 0.68 to 0.73. Although a slightly larger discrepancy in AUC values exists between these studies compared to ours, it is apparent that the variation in AUC values across all ML methods is not substantial. We attribute this lack of significant difference to the fact that both the training and validation cohorts were sourced from the same institution without external validation, a limitation we’ll address in our forthcoming sections.

An online calculator was created to facilitate individualized surgical treatment by calculating the risk for each patient of having a positive lymph node (https://wuqian.shinyapps.io/shinybsgi/). For instance, a women with the preoperative US showed a hyperechoic lymph node measuring 9*4 mm, with the CDFI grading absent, the tumour size of 14*10 mm, and the BSGI showed a negative mass in the axilla might be considered to have a approximately 16% risk of ALN metastasis, which implied that axillary surgery could be omitted clinically. Futhermore, the result in ROC analysis showed that the model could benefit from including BSGI features. To the best of our knowledge, no studies have incorporated BSGI features into the prediction model until now, however, the study result is limited and requires much more validation before it can be applied to clinical reasoning.

The advantage of BSGI lies in its combination of the physiological differentiation of the nuclear imaging and the anatomical differentiation of mammography. The semi-quantitative index TNR is traditionally applied in the diagnosis of breast cancer, with a value above 1.65 being considered to be highly suspicious [29], Studies have investigated the association between TNR and clinicopathological characteristics of breast cancer and found a positive correlation with the presence of ALN metastasis [30, 31], which is consistent with our study. In the comparison of the six ML approaches, we found that the tumour TNR was included in the NNET model, but unfortunately the SVM model was the final adoption. Nevertheless, there is no implication that the TNR value has no guidance in clinical practice. For the detection of metastatic ALN, studies showed the sensitivity of BSGI ranges between 67% and 100%, with an average of 81%, and the specificity between 64% and 100% [32]. A positive mass in the axilla indicates the possibility of ALN metastasis more graphically. Although obvious differences were shown in the importance of variables among six ML algorithms, axillary mass status held a relatively higher weight in all models.

US is a common preoperative imaging modality for breast cancer, previous studies have suggested that some US features may correlate with the status of ALN, such as cortical thickness of ALN, transverse diameter of ALN, and lymph node hilum status [8, 33]. In our study, both the transverse/longitudinal diameter of the tumour and the lymph node, the echogenicity of the lymph node, and the CDFI grade of the lymph node were all found to be statistically correlated with ALN status. The CDFI grade has been employed in the US to evaluate tumoral angiogenesis, which is a growing trend in breast cancer [34, 35]. Studies also showed that tumour vascularity was correlated with lymph node involvement [36], Chao [37] reported that ALN metastasis had a greater tendency to be present in carcinomas with neovascularisation based on an analyses of 368 patients. However, few researches have investigated the relationship between lymphatic CDFI grade and ALN burden, while our study obtained a positive correlation. Traditionally, hyperechogenicity of a mass are associated with benignity [38] and the same conclusion was reached in this study.

Compared with previous studies attempting to predict the risk of ALN metastases in breast cancer, our work has several strengths. First, few studies have included BSGI-related variables in the model, while a considerable number of studies have compared the specificity and sensitivity of BSGI with MRI and reached favourable conclusions, which confirms the rationality of BSGI as a preoperative modality. The ROC analysis in our study showed that using model with BSGI features provided additional benefit from only ultrasonographic features. Furthermore, all variables included were non-invasive, which might reduce the complications associated with invasive procedures such as SLNB. Finally, we applied ML approach and established an online calculator, which certainly provides the clinical decision making with greater convenience.

However, some limitations of the study were noted. Firstly, despite comparable accuracy to MRI, BSGI remains to be an uncommon modality owing to its high cost and radiation exposure, limiting the promotion of the model. What’s more, The relatively low sensitivity of the model reflected the inclusion of fewer patients with ALN metastases in the training data, a limitation that results in showing higher specificity in predicting the absence of ALN metastases. Finally, the model lacks valid external validation given that it was built and validated in the same institution and the nature of a retrospective study all might resulted in selection bias and a slight discrepancy in AUC.

## Conclusions

Overall, the trend of de-escalation of axillary surgery is inevitable. In this study, we sought to develop a non-invasive preoperative prediction model to facilitate the subsequent clinical management of patients using ML approach, and for the first time incorporated BSGI-related variables into the model in the hope of provoking subsequent researchers to explore the wider possibilities of BSGI in the management of breast cancer. This study revealed more areas that need future research to validate our findings.

### Electronic supplementary material

Below is the link to the electronic supplementary material.

Supplementary Material 1

## Data Availability

The datasets used and/or analysed during the current study are available from the corresponding author on reasonable request.

## Abbreviations

ALN: axillary lymph node
BSGI: breast specific gamma image
ML: machine learning
ROC: receiver operating characteristic
US: Ultrasonography
MRI: magnetic resonance imaging
DTs: decision trees
ANN: artificial neural networks
SVM: support vector machines
CC: Craniocaudal
MLO: mediolateral oblique
BIRADS: Breast Imaging Reporting and Data System
TNR: tumour-to-normal lesion ratio
ER: estrogen receptor
PR: progesterone receptor
LVI: lymphovascular invasion
SBR: Scarff-Bloom-Richardson
CDFI: color Doppler flow imaging
RI: resistance index
GLM: generalized linear model
RF: random forest
NNET: neural network
GBM: gradient boosting machine
SGB: extreme boosting machine
REF: recursive feature elimination
UOQ: Upper-outer quadrant
LOQ: Lower-outer quadrant
UIQ: Upper-inner quadrant
LIQ: Lower-inner quadrant
PPV: Positive predictive value
NPV: Negative predictive value
AUC: the area under the ROC curve
SLNB: sentinel lymph nodes biopsy
ALND: axillary lymph node dissection
