# Supplementary material for: A non-invasive preoperative prediction model for predicting axillary lymph node metastasis in breast cancer based on a machine learning approach: combining ultrasonographic parameters and breast gamma specific imaging features
Source: Radiat Oncol. 2024 May 27;19:63. doi: 10.1186/s13014-024-02453-2 (PMC11131273; doi:10.1186/s13014-024-02453-2)
Supplement: Supplementary file 1 — Supplementary Material 1 [file 13014_2024_2453_MOESM1_ESM.docx]

## Supplementary Materials

Table S1: Differences of clinicopathological characteristics between the training and test sets.

|  | Training set (n=235) | Test set (n=99) | All (n=334) | Pvalue |
| --- | --- | --- | --- | --- |
| **Gender** |  |  |  |  |
| female | 234 (99.6%) | 97 (98.0%) | 331 (99.1%) |  |
| male | 1 (0.4%) | 2 (2.0%) | 3 (0.9%) | 0.438 |
| **Age** |  |  |  |  |
| ≥56 | 136 (57.9%) | 55 (55.6%) | 191 (57.2%) |  |
| ＜56 | 99 (42.1%) | 44 (44.4%) | 143 (42.8%) | 0.787 |
| **Tumour location** |  |  |  |  |
| UOQ | 120 (51.1%) | 54 (54.5%) | 174 (52.1%) |  |
| non-UOQ | 115 (48.9%) | 45 (45.5%) | 160 (47.9%) | 0.644 |
|  |  |  |  |  |
| **BSGI features** |  |  |  |  |
| **Tumour TNR(CC)** |  |  |  |  |
| ≥2.5 | 124 (52.8%) | 49 (49.5%) | 173 (51.8%) |  |
| ＜2.5 | 111 (47.2%) | 50 (50.5%) | 161 (48.2%) | 0.67 |
| **Tumour TNR(MLO)** |  |  |  |  |
| ≥2.5 | 119 (50.6%) | 43 (43.4%) | 162 (48.5%) |  |
| ＜2.5 | 116 (49.4%) | 56 (56.6%) | 172 (51.5%) | 0.279 |
| **Axillary mass satus** |  |  |  |  |
| negative | 182 (77.4%) | 71 (71.7%) | 253 (75.7%) |  |
| positive | 53 (22.6%) | 28 (28.3%) | 81 (24.3%) | 0.329 |
|  |  |  |  |  |
| **Ultrasonic features** |  |  |  |  |
| **Tumour echogenicity** |  |  |  |  |
| low | 229 (97.4%) | 96 (97.0%) | 325 (97.3%) |  |
| other | 6 (2.6%) | 3 (3.0%) | 9 (2.7%) | 1 |
| **Transverse diameter of tumour (mm)** |  |  |  |  |
| ≥22 | 110 (46.8%) | 45 (45.5%) | 155 (46.4%) |  |
| ＜22 | 125 (53.2%) | 54 (54.5%) | 179 (53.6%) | 0.915 |
| **Longitudinal diameter of tumour (mm)** |  |  |  |  |
| ≥13 | 135 (57.4%) | 58 (58.6%) | 155 (46.4%) |  |
| ＜13 | 100 (42.6%) | 41 (41.4%) | 179 (53.6%) | 0.847 |
| **Tumour margin** |  |  |  |  |
| irregular | 234 (99.6%) | 96 (97.0%) | 330 (98.8%) |  |
| regular | 1 (0.4%) | 3 (3.0%) | 4 (1.2%) | 0.148 |
| **Tumour CDFI** |  |  |  |  |
| no signal (0) | 34 (14.5%) | 13 (13.1%) | 47 (14.1%) |  |
| spot (I) | 21 (8.9%) | 6 (6.1%) | 27 (8.1%) |  |
| linear (II) | 105 (44.7%) | 48 (48.5%) | 153 (45.8%) |  |
| abundant (III) | 75 (31.9%) | 32 (32.3%) | 107 (32.0%) | 0.795 |
| **Tumour RI** |  |  |  |  |
| 0 | 129 (54.9%) | 54 (54.5%) | 183 (54.8%) |  |
| >0 | 106 (45.1%) | 45(45.5%) | 151 (45.2%) | 0.953 |
| **Lymphatic echogenicity** |  |  |  |  |
| cystic | 84 (35.7%) | 32 (32.3%) | 116 (34.7%) |  |
| hypoechoic | 79 (33.6%) | 30 (30.3%) | 109 (32.6%) |  |
| hyperechoic | 68 (28.9%) | 37 (37.4%) | 105 (31.4%) |  |
| other | 4 (1.7%) | 0 (0.0%) | 4 (1.2%) | 0.292 |
| **Transverse diameter of lymph nodes (mm)** |  |  |  |  |
| no | 84 (35.7%) | 32 (32.3%) | 116 (34.7%) |  |
| ＜9.5 | 33 (14.0%) | 18 (18.2%) | 51 (15.3%) |  |
| ≥9.5 | 118 (50.3%) | 49 (49.5%) | 167 (50.0%) | 0.599 |
| **Longitudinal diameter of lymph nodes (mm)** |  |  |  |  |
| no | 84 (35.7%) | 32 (32.3%) | 116 (34.7%) |  |
| ＜5.1 | 51 (21.7%) | 25 (25.3%) | 76 (22.8%) |  |
| ≥5.1 | 100 (42.6%) | 42 (42.4%) | 142 (42.5%) | 0.733 |
| **Absence of lymph node hilum** |  |  |  |  |
| no or not described | 231 (98.3%) | 96 (97.0%) | 327 (97.9%) |  |
| yes | 4 (1.7%) | 3 (3.0%) | 7 (2.1%) | 0.722 |
| **Lymphatic CDFI** |  |  |  |  |
| no signal (0) | 177 (75.3%) | 72 (72.7%) | 249 (74.6%) |  |
| spot (I) | 12 (5.1%) | 5 (5.1%) | 17 (5.1%) |  |
| linear (II) | 31 (13.2%) | 13 (13.1%) | 44 (13.2%) |  |
| abundant (III) | 15 (6.4%) | 9 (9.1%) | 24 (7.2%) | 0.856 |
| **Lymphatic RI** |  |  |  |  |
| 0 | 225 (95.7%) | 92 (92.9%) | 317 (94.9%) |  |
| >0 | 10 (4.3%) | 7(7.1%) | 17 (5.1%) | 0.285 |
|  |  |  |  |  |
| **Pathological features** |  |  |  |  |
| **Infiltration depth** |  |  |  |  |
| in situ | 7 (3.0%) | 6 (6.1%) | 13 (3.9%) |  |
| infiltrative | 223 (94.9%) | 88 (88.9%) | 311 (93.1%) |  |
| other | 5 (2.1%) | 5 (5.1%) | 10 (3.0%) | 0.138 |
| **Histologic type** |  |  |  |  |
| ductal | 220 (93.6%) | 90 (90.9%) | 310 (92.8%) |  |
| lobular | 7 (3.0%) | 3 (3.0%) | 10 (3.0%) |  |
| other | 8 (3.4%) | 6 (6.1%) | 14 (4.2%) | 0.541 |
| **SBR grade** |  |  |  |  |
| no | 30 (12.8%) | 17 (17.2%) | 47 (14.1%) |  |
| I | 8 (3.4%) | 5 (5.1%) | 13 (3.9%) |  |
| II | 97 (41.3%) | 37 (37.4%) | 134 (40.1%) |  |
| III | 100 (42.6%) | 40 (40.4%) | 140 (41.9%) | 0.618 |
| **Estrogen receptor status** |  |  |  |  |
| negative | 57 (24.3%) | 32 (32.3%) | 89 (26.6%) |  |
| positive | 178 (75.7%) | 67 (67.7%) | 245 (73.4%) | 0.165 |
| **Progesterone receptor status** |  |  |  |  |
| negative | 80 (34.0%) | 44 (44.4%) | 124 (37.1%) |  |
| positive | 155 (66.0%) | 55 (55.6%) | 210 (62.9%) | 0.094 |
| **Proliferation index (Ki-67)** |  |  |  |  |
| ＜14% | 52 (22.1%) | 16 (16.2%) | 68 (20.4%) |  |
| ≥14% | 183 (77.9%) | 83 (83.8%) | 266 (79.6%) | 0.277 |
| **Her-2 overexpression** |  |  |  |  |
| negative | 172 (73.2%) | 73 (73.7%) | 245 (73.4%) |  |
| positive | 63 (26.8%) | 26 (26.3%) | 89 (26.6%) | 1 |
| **Molecular subtype** |  |  |  |  |
| Luminal A | 43 (18.3%) | 9 (9.1%) | 52 (15.6%) |  |
| Luminal B | 135 (57.4%) | 58 (58.6%) | 193 (57.8%) |  |
| Her2 positive | 27 (11.5%) | 18 (18.2%) | 45 (13.5%) |  |
| Triple negative | 30 (12.8%) | 14 (14.1%) | 44 (13.2%) | 0.101 |
| **lymphovascular invasion** |  |  |  |  |
| no | 206 (87.7%) | 79 (79.8%) | 285 (85.3%) |  |
| yes | 29 (12.3%) | 20 (20.2%) | 49 (14.7%) | 0.092 |
| **Multifocality** |  |  |  |  |
| no | 229 (97.4%) | 97 (98.0%) | 326 (97.6%) |  |
| yes | 6 (2.6%) | 2 (2.0%) | 8 (2.4%) | 1 |
| **T stage** |  |  |  |  |
| Tis | 7 (3.0%) | 6 (6.1%) | 13 (3.9%) |  |
| T1 | 122 (51.9%) | 51 (51.5%) | 173 (51.8%) |  |
| T2 | 98 (41.7%) | 38 (38.4%) | 136 (40.7%) |  |
| T3 | 8 (3.4%) | 4 (4%) | 12 (3.6%) | 0.579 |
| **N stage** |  |  |  |  |
| N0 | 143 (60.9%) | 60 (60.6%) | 203 (60.8%) |  |
| N1 | 48 (20.9%) | 21 (21.2%) | 70 (21.0%) |  |
| N2 | 28 (11.9%) | 10 (10.1%) | 38 (11.4%) |  |
| N3 | 15 (6.3%) | 8 (8.1%) | 23 (6.8%) | 0.919 |
| **Axillary lymph node metastasis** |  |  |  |  |
| yes | 92 (39.1%) | 39 (39.4%) | 131 (39.2%) |  |
| no | 143 (60.9%) | 60 (60.6%) | 203 (60.8%) | 1 |

UOQ: Upper-outer quadrant; BSGI: breast specific gamma image; TNR: tumour-to-normal lesion ratio; CC: craniocaudal; MLO: mediolateral oblique; CDFI: color Doppler flow imaging; SBR grade: Scarff-Bloom-Richardson grade.


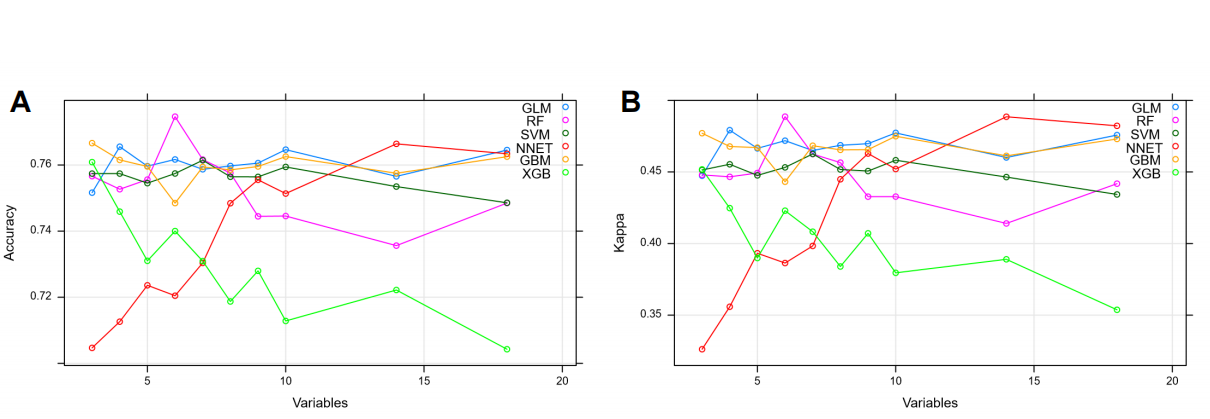


Figure S1: Variables selection with REF method. (A) The line graph shows the relationship between the number of candidate variables and the accuracy in each algorithm. (B) The line graph shows the relationship between the number of candidate variables and the Kappa values in each algorithm. GLM: generalized linear model; RF: random forest; SVM: support vector machine; NNET: neural network; GBM: gradient boosting machine; XGB: extreme boosting machine.

Table S2: The best hyperparameters of different machine learning models in evalution of variables importance

| **ML model** |  |  |  |  |  |  |  |
| --- | --- | --- | --- | --- | --- | --- | --- |
| GLM | alpha: 0 | lambda:0.5 |  |  |  |  |  |
| RF | ntree: 500 | mtry:1 |  |  |  |  |  |
| SVM | sigma: 0.5 | cost: 0.001 |  |  |  |  |  |
| NNET | size: 1 | decay: 0.1 |  |  |  |  |  |
| GBM | nums of trees:1000 | interaction depth: 3 | shrinkage: 0.01 | nums of minobsinnode: 5 |  |  |  |
| XGB | nrounds: 1000 | max depth: 20 | eta: 0.5 | gamma: 1 | colsample by tree: 1 | min child weight: 1 | subsample:0.8 |

ML: machine learning; GLM: generalized linear model; RF: random forest; SVM: support vector machine; NNET: neural network; GBM: gradient boosting machine; XGB: extreme boosting machine.
